# Integrating machine learning and GWAS for variant prioritization in the INCIPE cohort highlights ABC transporter genes in chronic kidney disease

**DOI:** 10.3389/fgene.2026.1902866

**Published:** 2026-08-31

**Authors:** Dagnogo Dramane, Mirko Treccani, Laura Veschetti, N’Guessan Benedicte Sonia Koffi, Cristina Patuzzo, Pietro Manuel Ferraro, Giovanni Gambaro, Dago Dougba Noel, Giovanni Malerba

**Affiliations:** 1 Department of Neurosciences, Biomedicine and Movement Sciences, University of Verona, Verona, Italy; 2 Department of Cellular, Computational and Integrative Biology, University of Trento, Povo (TN), Italy; 3 Unité de Formation et de Recherche (UFR) des Sciences Biologiques, Département de Biochimie Génétique, Université Peleforo Gon Coulibaly, Korhogo, Côte d’Ivoire; 4 Human Nutrition Unit, Department of Food and Drug, University of Parma, Parma, Italy; 5 Infections and Cystic Fibrosis Unit, Division of Immunology, Transplantation and Infectious Diseases, IRCCS San Raffaele Scientific Institute, Milano, Italy; 6 Vita-Salute San Raffaele University, Milano, Italy; 7 Division of Nephrology, Department of Medicine, University of Verona and Azienda Ospedaliera Universitaria Integrata, Verona, Italy; 8 GM Lab, Department of Surgical Sciences, Dentistry, Paediatrics and Gynaecology, University of Verona, Verona, Italy

**Keywords:** chronic kidney disease, genetic biomarkers, genome-wide association studies, imbalanced data, machine-learning, protein-protein interaction networks, SNP prioritization

## Abstract

**Introduction:**

Chronic kidney disease (CKD) is a major public health challenge, affecting approximately 674 million people worldwide and representing one of the fastest-growing causes of mortality. Since CKD is frequently asymptomatic in its early stages, the identification of novel genetic biomarkers may improve early detection and risk stratification. Genome-Wide Association Studies (GWAS) have identified numerous genetic loci associated with CKD and related traits; however, their performance is often limited in small and imbalanced cohorts, where reduced statistical power increases both false-positive and false-negative findings. Machine learning (ML) approaches can complement conventional GWAS by prioritizing biologically relevant genetic signals from high-dimensional genomic data.

**Methods:**

In this study, we implemented a nested ensemble (NCBC) model composed of an undersampler and a CatBoostClassifier (CBC) to prioritize candidate genetic variants associated with CKD in the INCIPE cohort. Prioritized variants were functionally annotated and evaluated through enrichment analyses, GTEx gene expression profiling, and protein-protein interaction network analyses. Genes identified by the CKDGen Consortium were analysed as an external reference set and used to validate the biological relevance of the prioritized results.

**Results:**

The NCBC model outperformed conventional ML classifiers, achieving a ROC AUC score of 87.77%, compared to 50%–53% for the other evaluated models. Among the prioritized genes, 56.25% showed protein-protein interactions with genes previously reported by the CKDGen Consortium, whereas only 1.9% of randomly generated gene sets showed interactions.

**Discussion:**

Our study demonstrates that the NCBC model improves the prioritization of biologically plausible candidate variants in a small and imbalanced CKD cohort. Functional analyses suggested ABC transporter-related genes, including ABCA13, ABCA4, and ABCC4 genes, as promising candidate for future validation, with ABCA4 showing substantial expression in kidney tissues. Overall, these findings support the integration of ML with GWAS to prioritize candidate genes and investigate the genetic architecture of complex diseases.

## 1 Introduction

Chronic kidney disease (CKD) is defined as the presence of abnormalities of kidney structure or function, present for more than 3 months, with implications for health (Author Anonymous, 2013). It is a major risk factor for premature mortality and increases the risk of developing other non-communicable diseases (NCDs) such as cardiovascular, respiratory, and metabolic disorders (O’Leary, 2024). CKD represents a major public health burden. Deaths attributable to CKD increased from 591,800 in 1990 to 1,425,670 in 2021 (Deng et al., 2025; Shahbazi et al., 2024), reaching 1.5 million deaths in 2021 (Deng et al., 2025), and are expected to rise to 2.2 million in the best-case scenario and 4 million in the worst-case scenario by 2040 (Foreman et al., 2018). Moreover, the WHO estimated that 674 million people were living with CKD in 2025 (World Health Organization, 2026). The silent characteristic of CKD makes most of the affected people unaware about their condition at the early stage, making prompt and effective intervention a challenge. Therefore, prioritizing the identification of biomarkers for CKD is essential for early detection and personalized treatments. The identification of biomarkers allows the identification of individuals at risk and provides healthcare professionals with critical tools to manage the disease more effectively (Kovesdy, 2022). In the past two decades, Genome-Wide Association Studies (GWAS) have emerged as primary methods for identifying SNPs associated with various diseases and traits (Visscher et al., 2017). The fundamental hypothesis behind a GWAS is that comparing the frequency of genetic variants between individuals presenting or not a particular trait or disease, we can identify Single Nucleotide Polymorphisms (SNPs) that are statistically associated with the trait. While this approach has contributed to the discovery of numerous disease-associated variants (Visscher et al., 2017), its efficacy is strictly dependent on the analysed samples, both in terms of size and distribution across the studied conditions. Generally, small sample sizes and imbalanced distribution dramatically decreases the statistical power of GWAS, hence increasing the risk of both Type I (false positive) and Type II (false negative) errors, making it inefficient (Politi et al., 2023).

Diverse approaches have been developed to address these issues, including machine learning (ML) methods. In the biology field, ML has been applied to a wide range of domains, including genomics, proteomics, and systems biology, and it aids in solving bioinformatic problems such as sequence similarity determination, pattern identification, and microarray data analysis. It is gaining incredible interest from health scientists (∼5,000 publications by year in 2015 to over 30.000 publications by year in 2023) (Machine Learning, 2026). However, this method presents some limitations, particularly when using drastic imbalanced data which significantly reduces the generalizability of the model (Öztornaci et al., 2023).

In this study, we combine an undersampling method with an ensemble ML model to enhance the predictive performance of the overall model using imbalanced and small sized CKD datasets of the Initiative on Nephropathy, of relevance to public health, which is Chronic, possibly in its Initial stages, and carries a Potential risk of major clinical End-points (INCIPE) cohort and explored CKD leading markers in this cohort.

## 2 Materials and methods

### 2.1 Data source and data extraction

The INCIPE study was carried out on 3,870 patients, all Caucasians, having at least 40 years old on 1 January 2006 (Gambaro et al., 2010). They were randomly chosen from the lists of patients of 62 randomly selected general practitioners based in four geographical areas in the Veneto region, Italy (Gambaro et al., 2010; Mallamaci and Tripepi, 2024). The GFR was estimated with the CKD Epidemiology Collaboration equation using calibrated creatinine (Levey et al., 2009). The definition of the five CKD stages was done according to the Kidney Disease Outcomes Quality Initiative (KDOQI) classification (Goolsby, 2002). Which determined 5 Stages to classified CKD patients from Stage 1 to Stage 5. Stage 3 was also sub-stratified into two classes: 3a: 45 < GFR 59 mL/min per 1.73 m^2^ with ACR ≥3.4 mg/mmol creatinine; and 3b: 30 < GFR 44 mL/min per 1.73 m^2^. The subjects were divided in two subgroups for genotyping: INCIPE1 using the Illumina Human Omni Express chip array (893 individuals, 650,655 SNPs) (Illumina Techniques, 2026) and INCIPE2 using the Illumina Human Core Exome chip array (2,153 individuals, 534,065 SNPs) (Guo et al., 2014; Boulygina et al., 2020; Santoro et al., 2023). We preprocessed both datasets using Plink standard quality control and then merged them. The merged dataset underwent genotype imputation (Treccani et al., 2023) on the Michigan Imputation Server, using the Haplotype Reference Consortium (HRC) panel. Variants showing an imputation confidence (R^2^ > 0.3) were retained. The resulting dataset comprised 3,046 individuals and 9,190,371 SNPs. To increase differences between cases and controls, we only considered Stage 1 CKD affected individuals as controls and severe patients (stages 4, 3B, and 3A having an albuminuria ≥30) as cases (Table 1). As defined by Levey et al. (2009), (stage 5,4,3B and 3A patients having an albuminuria ≥30 are considered as higher-risk individuals). The final data consisted of 213 cases and 1,525 controls for 9,190,371 markers.

**TABLE 1:** INCIPE data and its composition. 213 cases (stage 4 and 3 patients except stage 3a with albuminuria <30) and 1,525 controls (stage 1 patients).

​	Stage 1	Stage 3	Stage 4	Markers	Pval ≤ 0.05
1vs 4 + 3	1,525	213		9,190,371	81,684

### 2.2 Preprocessing for GWAS association

Genetic data underwent the following quality control, Minor Allele Frequency (maf >0.01), missing individuals (mind>0.01), missing genotyping rate (geno>0.01), and Hardy Weinberg Equilibrium (hwe>1.e−6) following PLINK standard quality control (QC). A GWAS was performed on quality-controlled data using PLINK2 version 2.0 (Chang et al., 2015). The association test has been performed using PLINK 2 generalized linear model parameter – glm we used as covariates the sex of patients and the first third PCs.

### 2.3 Preprocessing for ML models

We selected all the genetic position being nominally significant (p-value ≤ 0.05) in our GWAS. Selected features have been extracted from the original datasets using PLINK 1.9 (Purcell et al., 2007) using the PLINK standard quality control (QC) maf > 0.01, missing individuals (mind > 0.01), missing genotyping rate (geno > 0.01), and Hardy Weinberg Equilibrium (hwe >1.e−6)) plus the arguments – extract and – recode 12 with the flag compound-genotype. Compound-genotype produces an output in the genotype format 11,12,21, and 22, where 11 refers to homozygote for reference allele, 12 or 21 refers to heterozygote and 22 refers to homozygote for alternative allele. Genotype were converted from the 11, 12, 21, and 22 format to 1,2,3 format, where 2 referred to the heterozygotes genotypes 12 and 21; then, we applied a MinMaxscaler [X_scaled_ = X − X_min_/X_max_ − X_min_] function from the python module sklearn.preprocessing (version 1.0.2) (Pedregosa et al., 2011) to standardize the data. Moreover, we created two subsets of the data comprising the CKD status and (Feng et al., 2023) the feature data containing the genetic markers. The target data and the feature data have been used by the *train_test_split* function from Sklearn.model_selection to split the data into train data (80% of the whole data) and the test data (20% of the whole data) with the argument random_state = 42 to set the seed for reproducibility purposes.

### 2.4 Comparison of ML models

We compared our model to five standard classifier models: Light Gradient Boosting Machine (lc) (Ke et al., 2017), XGBoost Classifier (xbc) (Chen and Guestrin, 2016), Balanced RandomForest Classifier (bfr) (Lemaître et al., 2017), RandomForest Classifier (rf) (Pedregosa et al., 2011), and a CatBoostClassifier (cbc) (Prokhorenkova et al., 2017). The best parameters for each model have been determined by performing a 10 folds cross-validation on: depth or max_depth, testing all the possible values ranging between 3 and 10; learning_rate to adjust the model’s parameters, considering the following values: 0.01, 0.05, 0.1, 0.2, and 0.4; iterations or n_estimators to determine the optimal number of iterations, where we tested for 100 random values ranging between 10 and 1,500; and *l2_leaf_reg* to determine the best regulation factor, considering different values: 1, 3, 5, 7, 9. For this purpose, we used the function SearchCV from the Python module sklearn.model_selection version 1.0.2 (Pedregosa et al., 2011). For each classifier, we selected the best model from each model cross validation to build a final classifier and the Nested CatBoostClassifier (NCBC). The NCBC model used the same parameters of the CatBoostClassifier. Then, we tested the trained final models on the test data and retained their evaluation metrics for the comparison.

### 2.5 Nested Catboost Classifier (NCBC)

**FIGURE 1:**
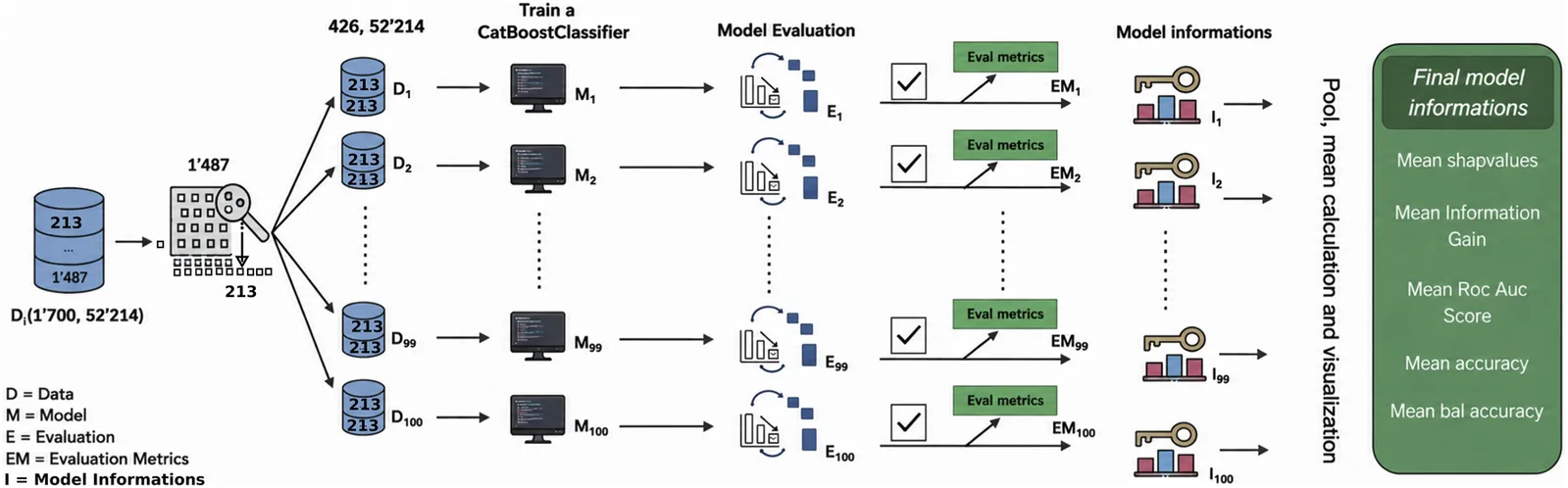
Nested CatBoostClassifier (NCBC). The model is composed a random undersampler which select 213 controls in each iteration *i* to form a balanced subset *Di* from the data *Dt*. This subset data is used to train a CatBoostClassifier *Mi* and then, the test data is used to evaluate it *Ei* and the model information’s are stored *Ii* for future use.

Catboost classifier was used to build the composed model with the best parameters obtained from the cross-validation (iterations = 200, l2_leaf_reg = 5, learning_rate = 0.2, depth = 8). NCBC is composed of a RandomUnderSampler from the Python module imblearn version 0.11.0 (Lemaître et al., 2017) which iteratively makes a random selection in the unaffected samples of the whole data *Dt* to fit its size with the affected samples size. The corresponding data set *Di* is used to train a new CatBoostClassifer (catboost 1.2.2) (Prokhorenkova et al., 2017). Then, the model is tested on the test data. Accurate models (roc_auc_score ≥ 75%) are selected where information regarding the features importance gain (FIG), Shapley Additive exPlanations (SHAP value) (shap version 0.44.0) (Lundberg and Lee, 2017), ROC AUC Score (roc_auc_score), accuracy, balanced accuracy (balanced_accuracy), True positive ratio (tpr) and False positive ratio (fpr) (sklearn.metrics 1.0.2) (Pedregosa et al., 2011) are extracted from the model evaluation and stored in a dedicated list. Moreover, the model is saved for future use. This scheme is repeated for 100 iterations in this work to ensure the selection of all the possible genetic structures. Finally, the mean of all the values is calculated, and the feature’s importance is ranked following their number of appearances as important for models (Figure 1).

### 2.6 Important SNPs assessment and annotation

Important SNPs have been assessed through the built-in CatBoostClassifier feature information gain (FIG ≥0.5) and SHAP value (SHAPvalue ≥0.05). The intersection of these features’ importances were annotated using the UCSC Variant Annotation Integrator tool (Hinrichs et al., 2016), using the human reference genome build GRCh37/hg19, and considering non-synonymous functional predictors (SIFT, PolyPhen2, MutationTaster), for known variants from the dbSNP database and their functional role (CDS, intergenic, regulator, etc.).

### 2.7 Functional analysis

**FIGURE 2:**
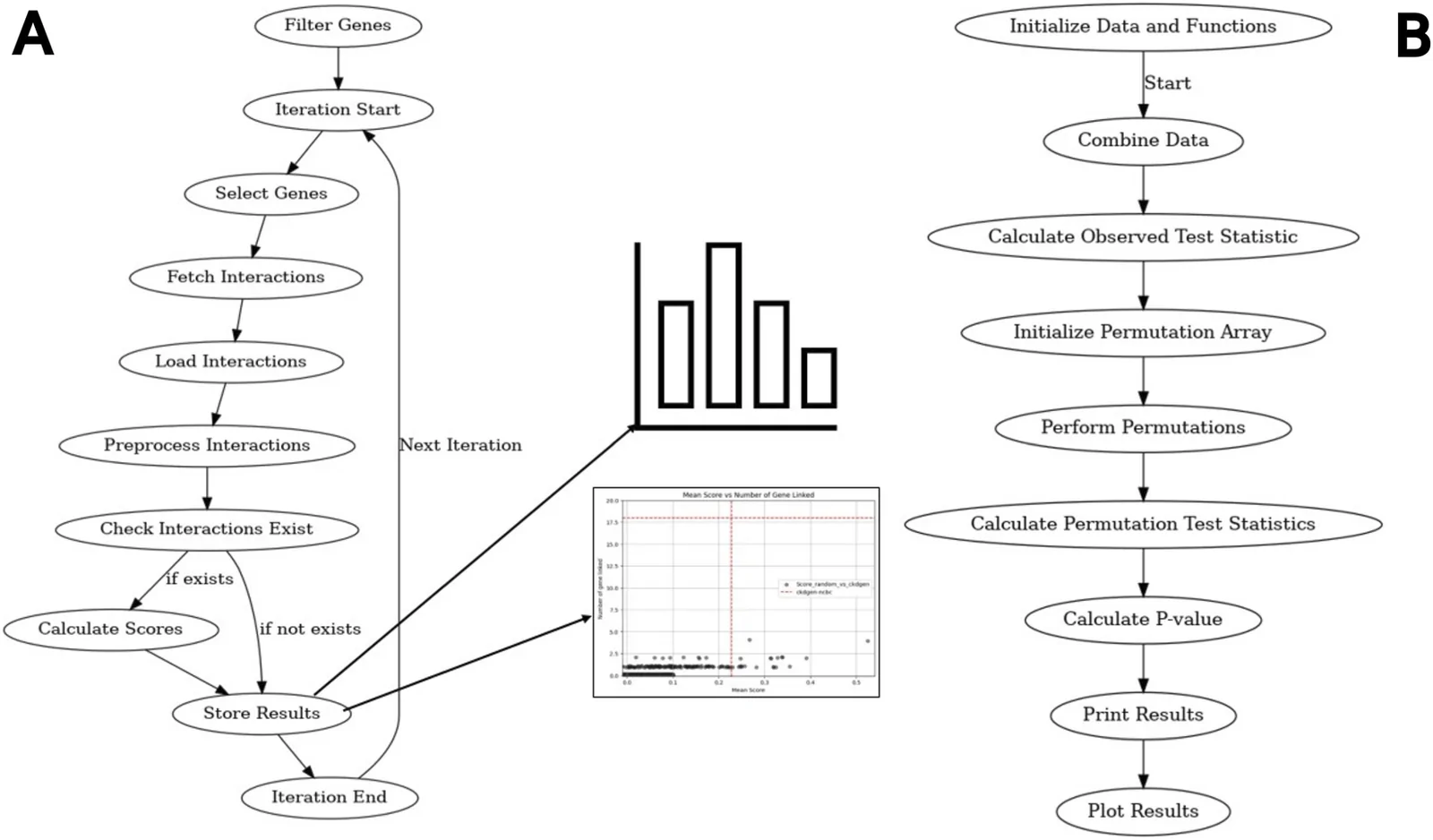
Workflow of the random genes-CKDGen Consortium genes graph analysis. **(A)** Random genes-CKDGen Consortium STRING graph analysis compared to NCBC genes -CKDGen consortium. **(B)** Permutation analysis.

We performed functional and gene sets enrichment analysis using g:Profiler (Raudvere et al., 2019) and its module Gene Ontology Statistics tool g:GOSt. We provide it with above mentioned genes from the annotation of the common SNPs (intersection of the two feature importance methods). So, we considered Gene Ontology terms from molecular function, cellular component, and biological process, and biological pathways from KEGG, Reactome, and WikiPathways, with a statistical threshold of adjusted by Benjamini–Hochberg p < 0.05. Following, we performed genes expression profile in GTEx version 8 (Lonsdale et al., 2013) in order to highlight any remarkable expression of these genes in the kidney cortex and kidney medulla tissues. Furthermore, we verified the presence of our genes in the GWAS Catalog (Buniello et al., 2019) and among the CKDGen Consortium articles (Köttgen and Pattaro, 2020). Finally we performed a gene network analysis using STRING version 12.0 (Szklarczyk et al., 2021) between the genes identified by the NCBC and the genes reported by the CKDGen Consortium; to support this analysis, we coupled it to a random graph analysis between the CKDGen Consortium genes and all the human coding genes from the annotation files of *Homo sapiens* Release 46 (GRCh38.p14) (Gencode, 2026). We first filtered the genes from the whole human genome to exclude those already present in the CKDGen Consortium genes and in NCBC selected genes. We then, conducted 10,000 iterations, each involving six steps which consisted of a Shahbazi et al. (2024) random selection of 33 genes as the number of genes in NCBC gene set from the filtered list; Feng et al. (2023) addition of the CKDGen Consortium genes to the selected genes; O’Leary (2024) using the fetch_string_interactions function (a custom function using STRING API) to obtain STRING interaction data for these genes; Kovesdy (2022) extracting from the interaction data co-expression scores, experimental determined scores, and curated database scores; Visscher et al. (2017) keeping the interactions involving only those containing the 33 selected genes; and (Politi et al., 2023) summarizing the interaction scores and the number of unique genes involved in each iteration. To clearly compare the average interaction scores with the number of linked genes and to highlight the key differences between human random genes and NCBC genes, we analysed the unique genes to determine how many random genes were linked to the CKDGen Consortium genes at each iteration. Then, the summarized interaction scores have been used to calculate the average interaction score of each connection (Figure 2A). Finally, we conducted permutation tests for the extracted scores (co-expression, experimental determined, and curated database scores), between NCBC and CKDGen, where NCBC scores represent the control group (group1) and the random genes scores represent the test group (group2). Due to the stochastic characteristic of this test, we set a random seed of 123 for reproducibility of the test. Each type of score values have been extracted from the two datasets. We performed the permutation test to determine if there was a significant difference between the two groups. For this, we implemented a custom function that involved calculating an observed test statistic, generating a distribution of permutation test statistics, and computing a p-value using NumPy (Harris et al., 2020) (Figure 2B).

## 3 Results

### 3.1 Genome wide association study

**FIGURE 3:**
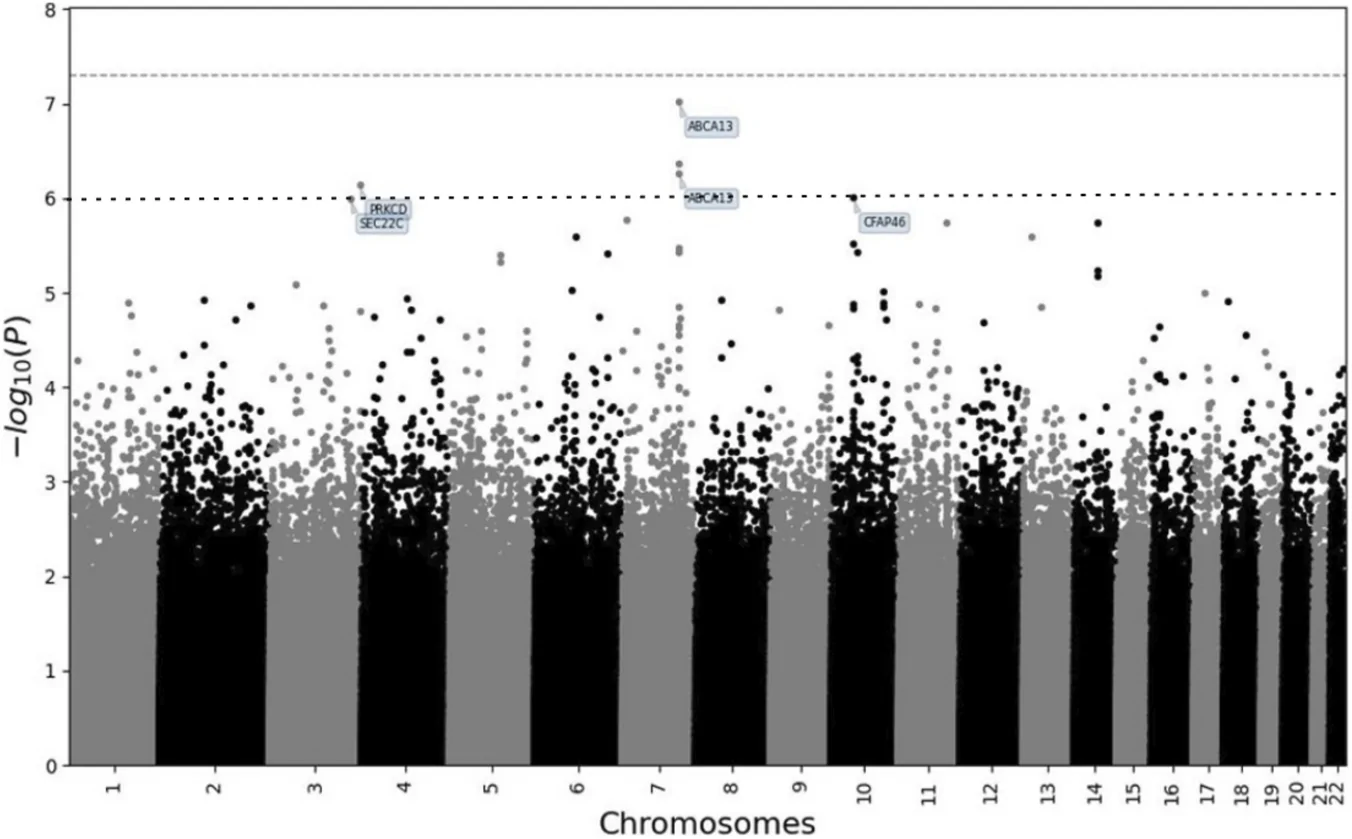
Manhattan plot of the association analysis of CKD in the INCIPE cohort. The most significant SNPs (p < 1 × 10^−6^) are reported together with the genes they belonged to, which have been annotated on the figure.

GWAS has been done to assess the potential risk SNPs associated with CKD and to perform feature selection from the GWAS summary statistics. Consistent with the limited sample size of the INCIPE cohort, no variant reached the genome-wide significance threshold (p-value > 5 × 10^−8^). To retain potentially informative signals for downstream integrative analyses, we applied a more tolerant significance threshold to p < 1 × 10^−6^, that identified four loci located in four different genes with strongest signals observed within ABCA13 (Figure 3). From this association summary, 81,684 markers that reached nominal significance (p-value ≤0.05) were selected for the NCBC investigation.

### 3.2 Machine learning models evaluation

**FIGURE 4:**
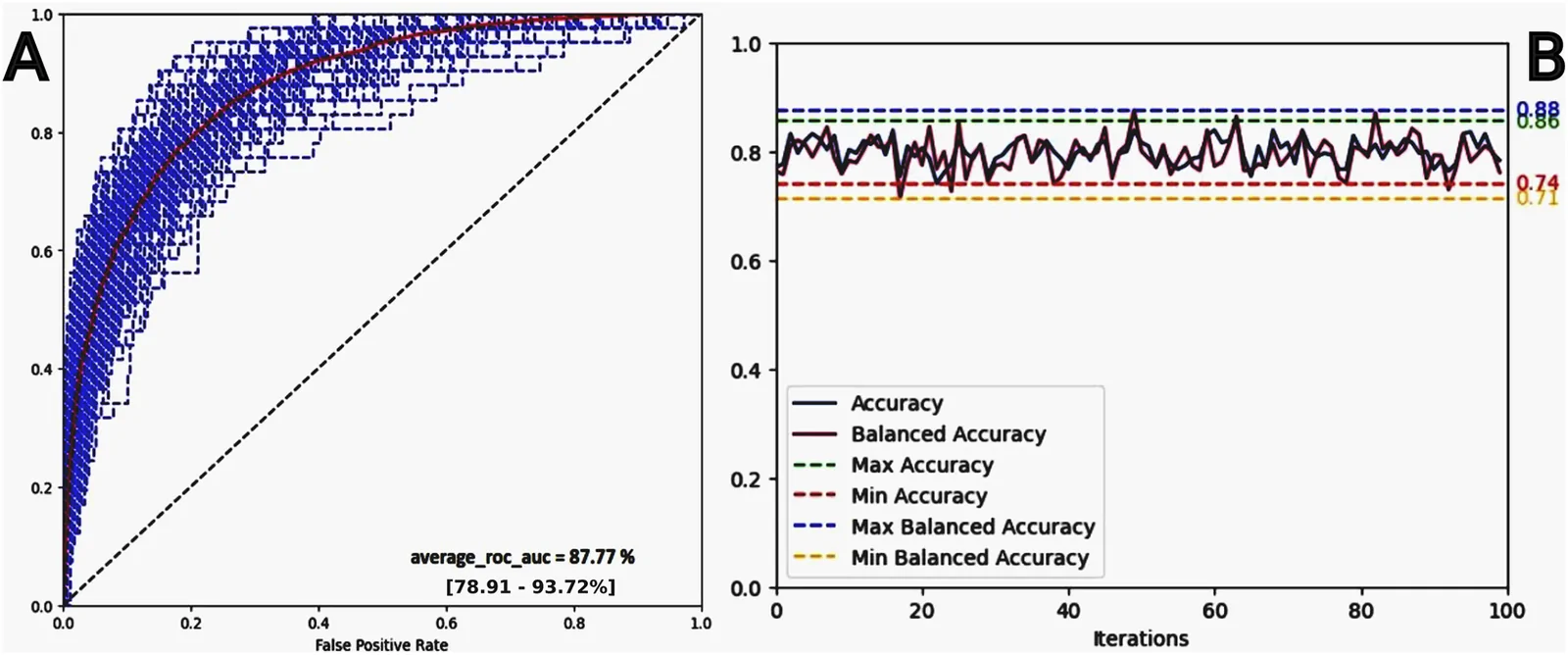
NCBC evaluation metrics on CKD in the INCIPE cohort. **(A)** ROC curve summarising 100 iterations curves; the red bold curve represents the mean ROC curve. **(B)** Accuracy and balanced accuracy across iterations: the red curve is the balanced accuracy evolution for each iteration and the blue curve is the accuracy curve. Dashed blue and green lines represent respectively the higher values of balance accuracy and accuracy while the dashed red and orange lines represent the low values of the accuracy and the balanced accuracy, respectively.

The performance of machine learning models was evaluated based on accuracy, balanced accuracy, the ROC AUC score, and computation time. The Ligth Gradient Boosting Machine classifier (lc) achieved an accuracy of 86.6, a balanced accuracy of 50, and an ROC AUC score of 50, with a computation time of 84.04 s. The eXtreme Gradient Boosting classifier (xbc) showed similar performance with an accuracy of 86.6, a balanced accuracy of 50, and an ROC AUC score of 50, but required 267.92 s (about 4 and a half seconds) to complete. The Random Forest classifier (rf) had an accuracy of 86.6, a balanced accuracy of 50, and an ROC AUC score of 50, with the shortest computation time of 1.46 s. The CatBoostClassifer (cbc), with a balanced accuracy of 53.47, and an ROC AUC score of 53.47, taking 44.34 s. Yet the performance of composed models rbf and NCBC are higher with a balanced accuracy of 78.51% and 79.95, a Roc auc score of 78.51% and 87.77% respectively for the rbf and NCBC (Table 2). However, NCBC proved to be the best model, achieving a ROC AUC score of 87.77 ± 0.029 (Figure 4A), the accuracy and the balanced accuracy confirmed this good prediction skill of our model on CKD phenotype of the INCIPE dataset with respectively percentages of 79.95 ± 0.023 and 79.45 ± 0.032. The worse model of the NCBC pool has reached a ROC AUC score value of 78.91% an accuracy of 74.18% and a balanced accuracy of 71.41% meanwhile the best model reached 93.72%, 85.62%, and 87.66% for the ROC AUC score, the accuracy, and the balanced accuracy, respectively (Figure 4B).

**TABLE 2:** The table reports the performance evaluation of the six models under investigation.

Classifiers	Time	Accuracy	Balanced accuracy	ROC AUC score
lc	84.04s	86.6	50	50
xbc	267.92s	86.6	50	50
bfr	36.32s	93.14	78.51	78.51
rf	1.46s	86.6	50	50
cbc	44.34s	87.25	53.47	53.47
NCBC	1.50h	79.95	79.95	87.77

Four metrics have been measured and compared: the training time, the accuracy of the model, the balanced accuracy and the roc auc score of the model. Lc, light gradient boosting machine classifier; xbc, eXtreme gradient boosting (xgboost) classifier; brf, balanced randomforest classifier; rf, randomforest classifier; ncbc, nested catboostclassifier; cbc, CatBoostClassifer. The grey box represents the best performance for each parameter.

### 3.3 Functional analysis of important SNPs

**FIGURE 5:**
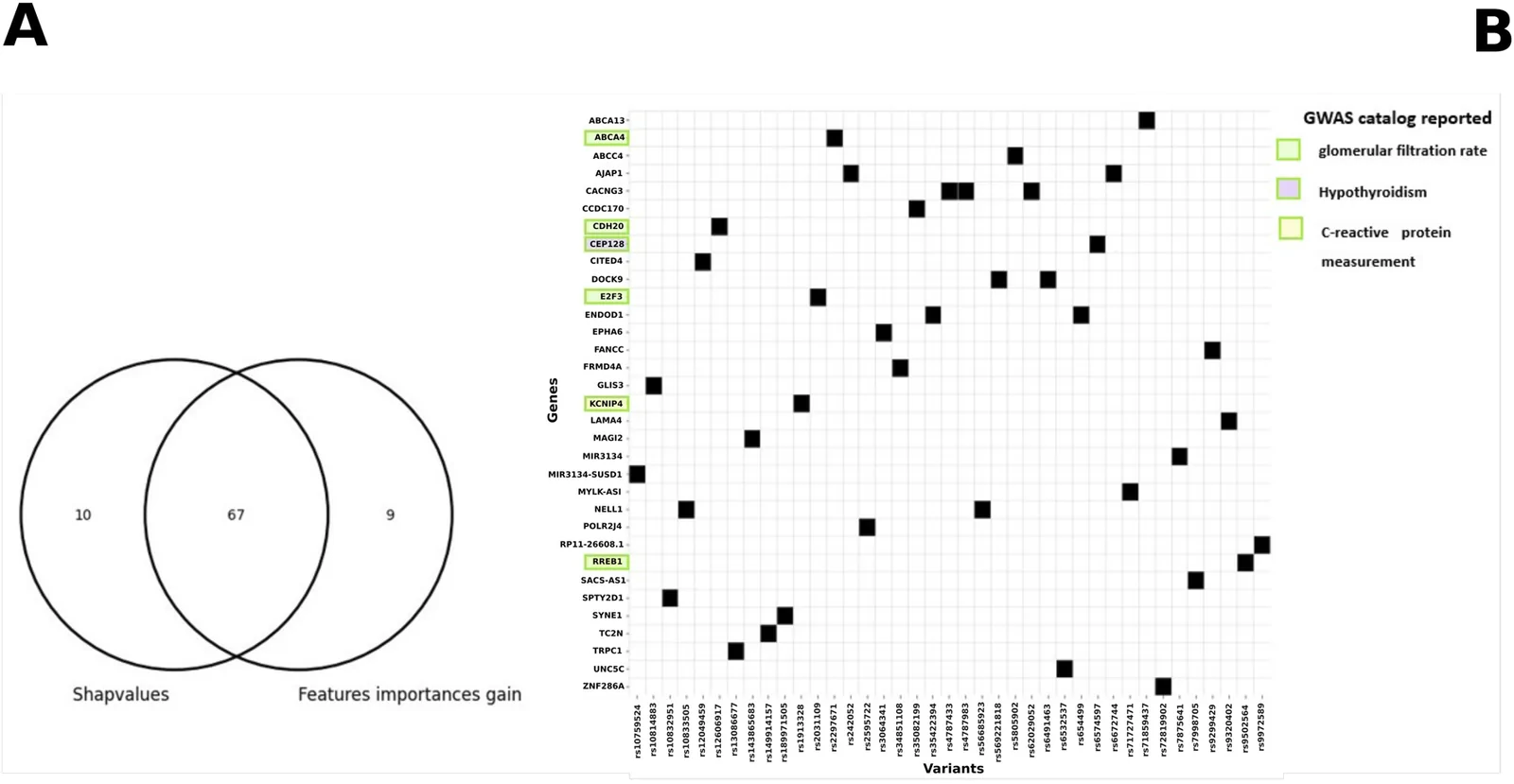
Important genes from the selected SNPs. **(A)** Venn’s diagram reporting the important common SNPs selected by features importance gain (FIG) and Shapvalues (SHAP) on INCIPE cohort. **(B)** Important genes reported in the GWAS Catalog as associated with traits related to CKD.

The NCBC model selected 76 SNPs having a FIG >0.5 and 77 SNPs having a SHAP value >0.05, sharing a total of 67 markers (Figure 5A). Annotation of these 67 SNPs resulted in 38 intronic variants located in 30 protein-coding genes (ABCA13, ABCA4, ABCC4, AJAP1, CACNG3, CCDC170, CDH20, CEP128, CITED4, DOCK9, E2F3, ENDOD1, EPHA6, FANCC, FRMD4A, GLIS3, KCNIP4, LAMA4, MAGI2, SUSD1, MYLK-AS1, NELL1, POLR2J4, SACS-AS1, SPTY2D1, SYNE1, TC2N, TRPC1, UNC5, and CZNF286A), 1 pseudogene (RP11O-266O8.1), 1 microRNA (miRNA, MIR3134) and 1 missense variant on the RREB1 gene. ABCA4, CDH20, E2F3, and RREB1 were already reported in the GWAS Catalog as associated with glomerular filtration rate (GFR), KCNIP4 with C-reactive protein measurement, and CEP128 with hypothyroidism (Figure 5B). Moreover, ABCA13, ABCA4, and ABCC4 are involved in ABC (ATP Binding Cassette) transporter pathways (KEGG:02010) (P-value ≤ 0.002), related to calcium transportation in different cells (Table 3). Among the identified candidates, ABCA4 emerged as particularly notable. Although classically considered a retina-associated gene, GTEx data revealed substantial expression in both kidney cortex and kidney medulla tissues, suggesting a previously underappreciated role in renal biology.

**TABLE 3:** Pathways related to important genes in the gene ontology and kegg databases.

Source	Term names	Term IDs	Adjusted p-values	Genes
GO:MF	ABC-type transporter activity	GO:0140359	0.009	ABCA4
GO:MF	ATPase-coupled transmembrane transporter activity	GO:0042626	0.0816	ABCA13
KEGG	ABC transporters	KEGG:02010	0.0013	ABCC4

P-values were adjusted for bh correction. Go, gene ontology; mf, molecular function; kegg, kyoto encyclopaedia of genes and genomes.

### 3.4 NCBC and CKDGen network interaction analysis

**FIGURE 6:**
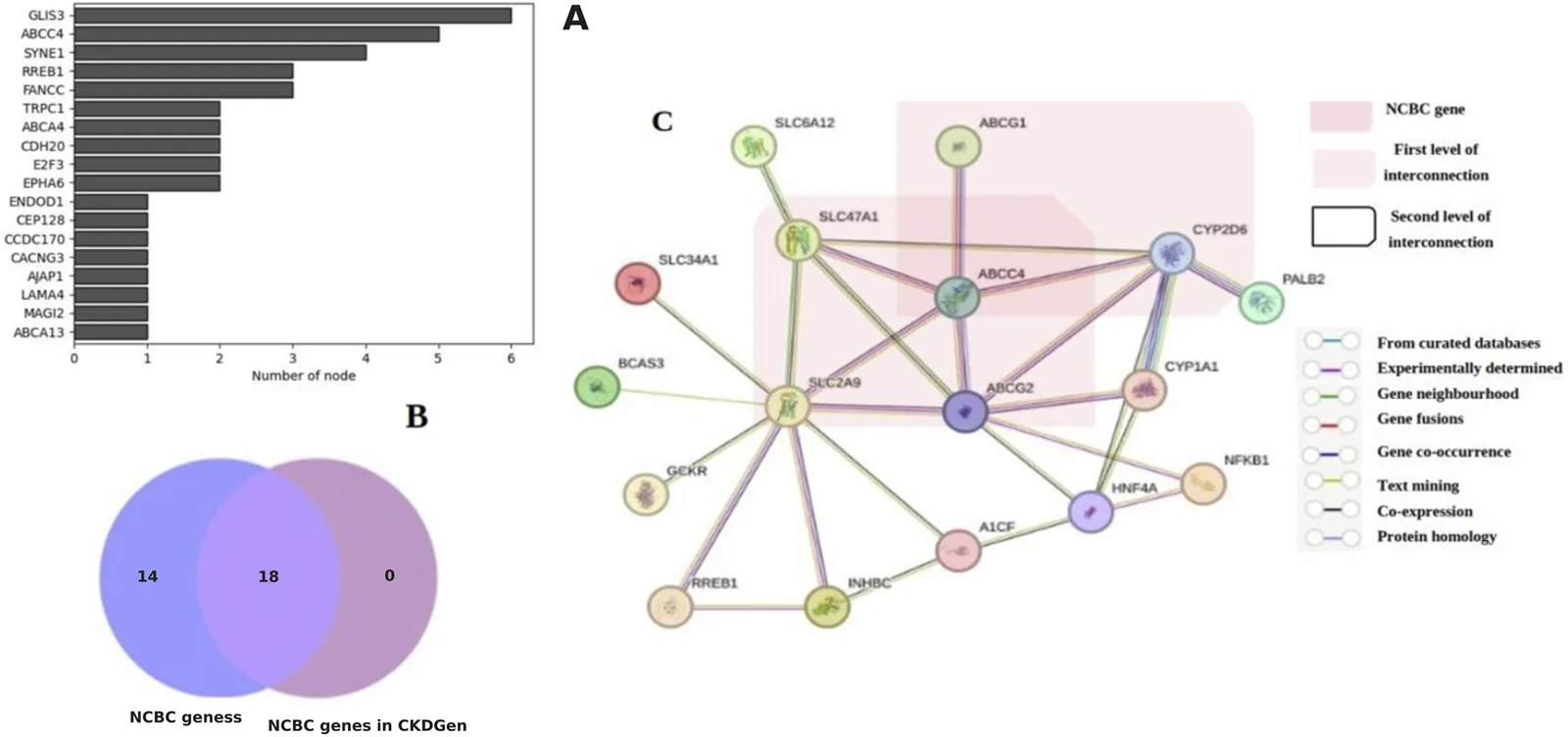
Shared interconnections between NCBC and CKDGen Consortium genes. **(A)** Number of CKDGen genes in the first-level connections with NCBC genes in the STRING network. **(B)** Total number of NCBC genes linked to at least one CKDGen Consortium gene in the venn diagramm intersection. **(C)** STRING network of first and second levels of CKDGen consortium proteins interaction with the NCBC gene ABCC4.

Our STRING protein-protein interaction analysis identified 18 NCBC genes exhibiting significant (pvalue <0.05) interconnections with the CKDGen Consortium genes (Figure 6B). Notably, genes such as ABCC4, GILS3, SYNE1, RREB1, and FANCC emerged as central nodes within the integrated network (Figure 6A; Supplementary Table S1). These genes are involved in essential biological pathways related to CKD and demonstrated significant interactions with well-established CKD biomarkers identified by the CKDGen Consortium. For example, ABCC4 was found to interact with ABCG2, ABCG1, SLC2A9, SLC47A1, and CYP2D6 (Figure 6C), which are recognized markers for CKD progression. Similarly, SYNE1 and RREB1 displayed substantial interactions with SOX17, INHBC, SLC2A9, LRP2, KMT2C, and PIP5K1B (Supplementary Table S1; Supplementary Figure S1). The graph of ABCC4 highlights an enrichment in small molecules transportation within the apical membrane, specifically being involved in biological processes related to coumarin and urate metabolism. It is also involved in molecular functions tied to the transmembrane transporter activity of urate and lipids, with 3 out of 5 genes involved in urate transport and 2 out of 8 genes involved in lipid transport. These findings collectively contribute to an enrichment in Monarch for hypouricosuria. SYNE1 and MAGI2 graphs are enriched in the End-Stage Renal Disease with 2 out of 5 genes and for conditions such as low molecular weight proteinuria, abnormal glomerular mesangium morphology, and nephrotic syndrome. RREB1 graph is enriched in small molecules transportation across the apical membrane of the proximal tubule and biological processes related to export across the plasma membrane, xenobiotic transport, and urate metabolism. LAMA4 and AJAP1 enriched in blood urea nitrogen, creatinine measurement, GFR, creatine measurement, and cystatin C measurement (Supplementary Table S1; Supplementary Figure S1).

**FIGURE 7:**
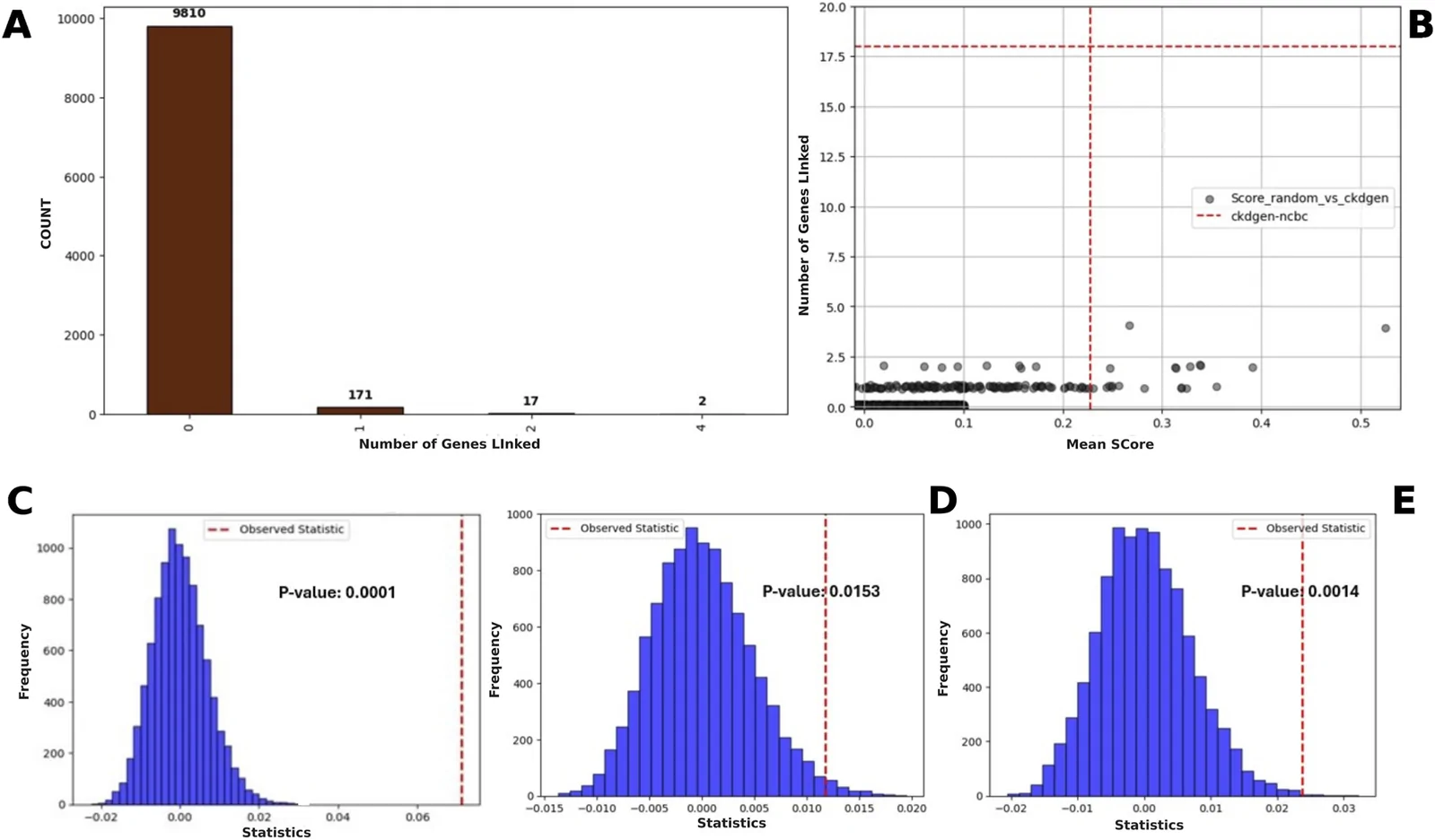
Protein–protein interaction analysis of NCBC genes and permutation-based comparison with randomly selected human coding genes. **(A)** Distribution of the number of randomly selected genes connected to at least one CKDGen Consortium gene across 10,000 random gene sets. Each iteration consisted of a randomly selected set of 33 human protein-coding genes (matching the number of NCBC genes) drawn from all annotated human coding genes after excluding genes previously identified by the NCBC and CKDGen Consortium. The y-axis shows the number of permutations yielding the corresponding number of linked genes. **(B)** Mean STRING protein–protein interaction scores for each random gene set plotted against the number of genes connected to CKDGen Consortium genes. Interaction scores were calculated using STRING v12.0 and included evidence derived from curated databases, experimentally determined interactions, and co-expression. Each point represents one of the 10,000 random gene sets. The red dashed lines indicate the observed values obtained for the NCBC gene set (18 linked genes and mean interaction score of 0.23). **(C–E)** Permutation test results comparing the observed interaction scores of the NCBC gene set with the null distribution generated from the 10,000 random gene sets. Histograms show the distribution of permutation test statistics, while the red dashed vertical line represents the observed statistic. **(C)** Curated database interaction score (P = 0.0001). **(D)** Experimentally determined interaction score (P = 0.0014). **(E)** Co-expression interaction score (P = 0.0153).

The random genes protein-protein interaction analysis showed only 1.9% of gene sets having at least one interaction with the CKDGen Consortium genes. Among them, only 0.16% have a mean score greater than or equal to the NCBC genes mean score (0.22) (Figure 7B). Moreover, the number of genes linked to CKDGen network in each gene sets was very low with (1) four genes in two gene sets, (2) two genes in 17 gene sets, and (3) only one gene in 171 gene sets out of 10,000 random gene sets from all human coding genes excluded NCBC and CKDGen genes; meanwhile, the NCBC gene set have 18 genes that shared at least one node with the CKDGen Consortium genes network (Figure 7A). The permutation analysis ensured the validity of our results: the Curated database interaction score displayed a strong significant p-value <0.01 (Figure 7C); concordantly, the experimental determined interaction score and the co-expression interaction score were significant, with a p-value of 0.0014 and 0.0153, respectively (Figures 7D,E). The observation that more than half of NCBC-prioritized genes are embedded within known CKD-related interaction networks suggests that the model captures biologically meaningful signals rather than random statistical artifacts.

## 4 Discussion

This study used a new machine learning approach to enhance signals identification in low and imbalanced CKD data. The GWAS results obtained in this analysis did not reach genome-wide statistical significance (p < 5 × 10^−8^). This lack of significance may be attributed to numerous factors, such as sample size or the distribution of traits across samples. Manolio and colleagues (Manolio et al., 2009) reported that studies with low sample sizes will have difficulties detecting rare variants or variants with small effects, which results in the low power of GWAS for complex diseases. Our implemented approach significantly improved the accuracy of the classification from 53% in the simplest model (cbc) to 87.77% in the NCBC model. These results suggest that machine learning based prioritization can complement traditional GWAS approaches by identifying biologically coherent signals that may not achieve genome-wide significance in low sized cohorts. Improved accuracy of the classification leads to a better identification of significantly associated signals, leading to the discovery and validation of candidate variants and genes, the identification and validation of biological pathways involved in CKD, providing a more comprehensive understanding of the disease aetiology (Visscher et al., 2017). Our investigation resulted in the significant association of some genes related to kidney traits and functions according to the GWAS Catalog. The genes ABCA4, CDH20, E2F3, and RREB1 have been previously reported to be associated with glomerular filtration rate (GFR), KCNIP4 was reported to be associated with C-reactive protein measurement, and CEP128 with hypothyroidism. These traits are well known CKD biomarkers, especially GFR, which is the gold standard for CKD diagnosis. This consistency with existing literature reinforces the validity of our findings and suggests that these genes may play a crucial role in the disease. However, none of the NCBC genes have been reported in CKDGen Consortium so far. STRING protein-protein interaction network analysis between the NCBC and CKDGen genes, resulted that 18 out of the 32 (we excluded the miRNA since they are not reported in STRING database) NCBC genes were directly connected to at least one gene reported in the CKDGen Consortium. The random graph analysis supports this result with only 180 out of 10,000 gene sets (1.9%) showng a link with the graphs of CKDGen Consortium genes, out of which 0.16% have a mean score ≥0.22 (mean score of NCBC genes). The maximum genes linked to the CKDGen Consortium were four genes in 2 out of 10,000 gene sets, which were very low compared to NCBC genes where 56.25% (Chang et al., 2015) are sharing at least one graph with the CKDGen Consortium genes. This result confirmed the validity of the NCBC results and the confirm the capability of that model to deal with low sample size and imbalanced data of complex diseases. Furthermore, the permutations analysis on the main scores of STRING showed significant differences between NCBC-CKDGen Consortium and random genes-CKDGen Consortium for the interactions in curated databases, experimentally determined, and co-expression with p-value ≤0.01. Although none of prioritized genes have yet been reported by the CKDGen Consortium, more than half were directly connected to established CKD associated genes through STRING interaction networks, and this connectivity was significantly greather than expected by chance according to both random-network and permutation analysis. These observations suggest that the identified genes may represent biologically relevant signals that remain undetected by conventional association studies in small size cohorts. Moreover, GLIS3, ABCC4, and SYNE1 genes respectively share six, five, and four protein-protein interactions with CKDGen Consortium genes. They seem to play a key role in their network organization and may be involved in common biological pathways or processes related to CKD like toxins and urate elimination. The STRING interaction network of ABCC4 highlights an enrichment in renal mechanisms including small molecules transportation within the apical membrane, specifically involving biological processes related to coumarin, urate metabolism, transmembrane transporter activity of urate, and lipids, with 3 out of 5 genes involved in urate transport and 2 out of 8 genes involved in lipid transport. These findings collectively contribute to an enrichment in Monarch for hypouricemia that has been reported to be associated with an increase in the risk for renal function deterioration (Manolio et al., 2009). SYNE1 and MAGI2 graphs are enriched in the End-Stage Renal Disease with 2 out of 5 genes and for conditions such as low molecular weight proteinuria, abnormal glomerular mesangium morphology, and nephrotic syndrome. This enrichment highlights the potential implication of these genes in severe CKD. Zhu et al. (2019) found that patients with MAGI2 mutations typically present severe nephrotic syndrome in early childhood, characterized by significant proteinuria, oedema, and hypoalbuminemia. They concluded that the early onset and severity of symptoms underscore the critical role of MAGI2 in kidney health. Furthermore, pathogenic mutations in MAGI2 have been convincingly linked to Congenital Nephrotic Syndrome (Bierzynska et al., 2017; Ashraf et al., 2018), highlighting the gene’s importance in renal development and disease. RREB1 connections are enriched in small molecules transportation across the apical membrane of the proximal tubule and biological processes related to exportation across the plasma membrane, xenobiotic transport, and urate metabolism. NCBC genes protein-protein interaction analysis highlight that these genes play a crucial role in the development and progression of CKD. Through their interactions and shared pathways with the CKDGen Consortium genes, they seem to contribute to various aspects of CKD physiopathology, including, glomerulosclerosis, cellular metabolism, excretory and transportation activity. Finally, among all identified genes, ABCA4 deserves particular attention. Although predominantly studied in retinal disorders such as Stargardt disease (Molday, 2015; Molday et al., 2022), GTEx expression data indicate substantial expression in renal tissues. Together with the enrichment of ABC transporter pathways observed in this study, this finding raises the possibility that ABCA4 contributes to renal transport processes and CKD susceptibility. Functional studies will be required to validate this hypothesis.

Several limitations should be considered when interpreting our findings. First, the candidate variants and genes identified in this study were derived from a single cohort and have not be replicated in another CKD dataset. So, external validation will be necesary to assess the generazability of the identified biomarkers. Second, the biological relevance of the prioritized genes was infered using functional enrichment analyses, GTEx expression data, and protein-protein interaction networks. While the convergence of these complementary approaches supports the robutness of our findings, these analyses provide indirect evidence and do not establish causal relationships. Finally, functional experiments were beyond the scope of this work. Therefore additionnal molecular and physiological studies will be required to determine the precise role of the identified genes, particularly ABC transporter family members such as ABCA4, ABCA13, and ABCC4, in CKD development and it’s progression.

## 5 Conclusion

By integrating GWAS and ML-based prioritization, this study supported the potential of combining complementary computational approaches in relatively small and imbalanced cohorts to identify novel plausible candidate genes and related pathway associated with complex conditions, such as CKD. The NCBC approach prioritized 67 variants corresponding to 32 genes, among which more than half were connected to established CKD loci previously identified by the CKDGen Consortium; this supported the biological relevance of the prioritized signals and the overall robustness of the proposed approach. Among the prioritized genes, enrichment analysis showed hints of association with ABC transporter pathway, suggesting a potential contribution of ABCA13, ABCA4, and ABCC4 in CKD susceptibility, as for the suggested downregulation of ABCA4 in CKD kidney tissues. Overall, these findings provide new insights into CKD genetic architecture and generate novel testable hypothesis for future functional and translational studies.

## Data Availability

The data analyzed in this study are subject to access restrictions and require approval from the Nephrology Unit of the University of Verona. Requests to access these datasets should be directed to dramane.dagnogo@univr.it.
