# Supplementary material for: Integrating machine learning and GWAS for variant prioritization in the INCIPE cohort highlights ABC transporter genes in chronic kidney disease
Source: Front Genet. 2026 Aug 31;17:1902866. doi: 10.3389/fgene.2026.1902866 (PMC13571820; doi:10.3389/fgene.2026.1902866)
Supplement: Supplementary file 1 [file Supplementaryfile1.docx]

**Supplementary material**

Fig s1. STRING graph of NCBC genes showing at least one interconnection with CKDGen consortium genes: A. E2F3. B. CEP128. C. EPHA6. D. RREB1. E. ENDOD1. F. CCDC170. G. LAMA4. H. MAGI2. I. GILS3. J. CDH20. K. AJAP1. L. SYNE1. M. CACNG3. N. TRPC1. O. ABCA4. P. FANCC. Q. ABCA13.

Table S1. Summary of GWAS results for the INCIPE CKD phenotype. Variants reaching the predefined flexible significance threshold (P < 1 × 10⁻⁶) are shown.

| **CHR** | **POS** | **ID** | **REF** | **ALT** | **A1** | **OBS_CT** | **OR** | **LOG(OR)_SE** | **Z_STAT** | **P** | **Genes** |
| --- | --- | --- | --- | --- | --- | --- | --- | --- | --- | --- | --- |
| 3 | 42596488 | rs6766225 | C | A | A | 1528 | 2.0536 | 0.147084 | 4.8924 | 9.9613E-07 | SEC22C |
| 3 | 53195120 | rs141359357 | G | A | A | 1528 | 3.03111 | 0.223603 | 4.95936 | 7.07247E-07 | PRKCD |
| 7 | 48661286 | rs17663162 | T | C | C | 1528 | 1.8596 | 0.12382 | 5.01019 | 5.4376E-07 | ABCA13 |
| 7 | 48668030 | rs1918582 | C | T | T | 1528 | 2.70816 | 0.186738 | 5.33512 | 9.54793E-08 | ABCA13 |
| 7 | 48696458 | rs4244230 | A | G | G | 1528 | 2.06663 | 0.143658 | 5.05312 | 4.34653E-07 | . |
| 7 | 48696943 | rs11771405 | A | G | G | 1528 | 2.06663 | 0.143658 | 5.05312 | 4.34653E-07 | . |
| 7 | 48728580 | rs140471798 | CA | C | C | 1528 | 2.01726 | 0.142726 | 4.91671 | 8.8013E-07 | . |
| 10 | 134633353 | rs4880277 | G | A | A | 1528 | 1.68176 | 0.106181 | 4.89577 | 9.79216E-07 | CFAP46 |

*Table s2*: Interconnections between NCBC genes and CKDGen consortium genes.

|  | **First Node** | **Second Node** | **Third Node** |
| --- | --- | --- | --- |
| **E2F3** | NFKB1, TFDP2 | NFATC1, TGFB1, JAG1, PIP5K1B, SCARB2, TRPC1, TMEM60, ABCG2, HNF4A, NOD2, RRM2, IRF5, CASP9, ATXN2, PIK3R1, MUC1 | ANXA9, WNT7A, DAB2, SKIL, KMT2C, ABCG1, LRP2, TBX2, NPHS1, CYP1A1, RET, SLC6A12, CST3, RHOC, USF2, SYNE1, NFKB1, PKD2, DDX1, TFDP2, SOS2, SLC2A9, NPPA, SLC6A13, HNF1A, CYP2D6, PLCE1, TGFB1, NTN1, GALNTL5, MED1, COL18A1, PELI1, ETV5, IGFBP5, NPPB, A1CF, PSTPIP1, ATXN2L, ABCC4, UBE2Q2, SOX17, WDR72, PHTF2, PRKCI, SIGIRR, FAM122A, SLC47A1, GALNT11, INHBC, WDR37 |
| **CEP128** | CEP89 | 0 | 0 |
| **EPHA6** | EFNA3, HPCAL1 | NTN1 | NFATC1, RET, EFNA3, ROBO2 |
| **RREB1** | SLC2A9, SOX17, INHBC | ABCG2, DAB2, TGFB1, HNF4A, GCKR, SLC47A1, ROBO2, SLC34A1, A1CF, ABCC4, BCAS3 | WNT7A, NFATC1, SKIL, ABCG1, LRP2, PFN3, NPHS1, CYP1A1, SCARB2, STC1, CST3, USF2, SCARB2, NFKB1, NFKB1, SLC2A9, NPPA, HNF1A, CYP2D6, SHROOM3, GATM, NTN1, MED1, KBTBD2, COL18A1, PIK3R1, JAG1, RGS14, IGFBP5, NPPB, CUBN, UBE2Q2, WDR72, KCNQ1, SLC7A9, IRF5, TBX2, CASP9, FNDC4, GLIS3, MUC1 |
| **ENDOD1** | LMAN2 | PRR7 | 0 |
| **CCDC170** | SYNE1 | PIP5K1B, LRP2, KMT2C | ACP1, DAB2, PLCE1, TFDP2, FAM122A, NPHS1, SYNE1, SLC34A1, NSD1, SCARB2, CUBN, CST3, PALB2, PIK3R1 |
| **LAMA4** | WDR72 | PDILT, TMEM60, WDR37, BCAS3 | ANXA9, PHTF2, SLC6A13, SLC2A9, TBX2, GLIS3, UNCX, TFDP2 |
| **MAGI2** | NPHS1 | PTPRO, TGFB1, LRP2, PLCE1, ROBO2, NPPB, CUBN, CST3, PIK3R1 | NFATC1, DAB2, SKIL, KMT2C, NPHS1, SCARB2, RET, CST3, RHOC, SCARB2, SYNE1, SLC34A1, NFKB1, HNF4A, SOS2, UNC5CL, NPPA, TGFB1, CST9, NTN1, MMACHC, COL18A1, ARL15, JAG1, IGFBP5, PIP5K1B, SOX17, ACP1, SLC7A9, IRF5, PRKCI, CASP9, INHBC, MUC1 |
| **GLIS3** | KCNQ1, JAZF1, EXD1, TDRD12, HNF1A, BCAS3 | WDR72, SLC2A9, SKIL, NPPA, RPL3L, HNF4A, PRKAG2, TBX2, A1CF, GCKR, MED1 | WNT7A, TGFB1, FNDC4, TMEM60, TBX2, CYP1A1, UBE2Q2, RREB1, PALB2, SLC34A1, NFKB1, DDX1, SLC2A9, HNF4A, JAZF1, CYP2D6, TSC1, KDM5A, JAG1, NSD1, KBTBD2, CDK12, OSTN, UNCX, JAG1, GCKR, PDILT, USF2, NPPB, RRAGD, ABCC4, UBE2Q2, SOX17, ABCG2, SYPL2, PRKCI, USF2, FAM122A, SLC47A1, LAMA4, INHBC, CST3, ALMS1, WDR37 |
| **CDH20** | CDH23, RNF152 | EYA4, ABCA4 | DACH1, TTC8 |
| **ABCC4** | SLC2A9, SLC47A1, ABCG1, CYP2D6, ABCG2 | NFKB1, HNF4A, PALB2, GCKR, CYP1A1, INHBC, SLC6A12, A1CF, RREB1, SLC34A1, BCAS3 | WNT7A, TGFB1, FAAP24, LRP2, PFN3, UBE2Q2, USF2, NFKB1, KMT2C, RPL3L, HNF1A, KDM5A, SHROOM3, GATM, MED1, RREB1, KBTBD2, CDK12, FANCC, JAG1, RGS14, NFATC1, SOX17, WDR72, ACP1, KCNQ1, RECQL4, MED1, TBX2, FNDC4, GLIS3, STC1 |
| **SYNE1** | CCDC170, KMT2C, PIP5K1B, LRP2 | ACP1, DAB2, TFDP2, PLCE1, FAM122A, NPHS1, SCARB2, SLC34A1, NSD1, CUBN, CST3, PALB2, PIK3R1 | TGFB1, ABCG1, FAAP24, PFN3, RET, STC1, HOXD11, RHOC, SCARB2, NFKB1, SLC2A9, HNF4A, SOS2, UNC5CL, SLC2A9, RPL3L, NPPA, RRM2, HNF1A, TGFB1, KDM5A, SHROOM3, CST9, GATM, MED1, KBTBD2, MMACHC, CDK12, FANCC, PTPRO, RGS14, ROBO2, NPPB, TMEM60, UBE2Q2, SOX17, E2F3, RECQL4, MAGI2, SLC7A9, CYP2D6, PRKCI, PRKAG2, INHBC, MUC1 |
| **CACNG3** | CACNA1S | SYPL2 | KBTBD2, UNCX, SKIL |
| **TRPC1** | NFKB1, PKD2 | ABCG2, E2F3, TGFB1, JAG1, HNF4A, NOD2, NFATC1, IRF5, IFT172, CASP9, SCARB2, ATXN2, PIK3R1, MUC1 | DAB2, WNT7A, SKIL, KMT2C, ABCG1, LRP2, TBX2, NPHS1, CYP1A1, RET, SLC6A12, CST3, RHOC, TTC8, USF2, NFKB1, DDX1, SOS2, SLC2A9, NPPA, HNF1A, CYP2D6, PLCE1, TGFB1, NTN1, GALNTL5, MED1, COL18A1, PELI1, ETV5, SDCCAG8, IGFBP5, PIP5K1B, WDPCP, TFDP2, NPPB, A1CF, ATXN2L, ABCC4, UBE2Q2, SOX17, PRKCI, SLC47A1, GALNT11, INHBC, FNDC4, ALMS1, PKD2 |
| **ABCA4** | CDH23, TTC8 | IFT172, SDCCAG8, WDPCP, CDH20, EYA4, MKKS, ALMS1 | , PKD2, ASTN2, SLC7A9, PRKAG2, NAT8, DACH1, FNDC4, RNF152 |
| **AJAP1** | UNCX | SKIL, SYPL2, PDILT, SHROOM3, KBTBD2, IGFBP5 | KBTBD2, UNCX, TGFB1, AP5B1, WNT7A, SPATA5L1, CACNA1S, SLC7A9, PRKCI, BCAS1, GATM, A1CF, JAZF1, SLC34A1, WDR72 |
| **ABCA13** | GALNTL5 | MUC1 | TGFB1, GALNT11, SCARB2, GALNTL5, NFKB1, COL18A1 |
| **FANCC** | PALB2, RECQL4, FAAP24 | ACP1, PALB2, RPL3L, CYP2D6, KDM5A, ORC4, DDX1, CDK12, KMT2C | FBXL20, SETDB1, PFN3, CYP1A1, SYNE1, SLC2A9, HNF4A, HNF1A, NSD1, MED1, KBTBD2, PIK3R1, FANCC, TDRD12, ABCC4, UBE2Q2, ABCG2, KCNQ1, SLC47A1, INHBC |

*Table s3*: Gene ontology of Interconnected genes between NCBC and CKDGen consortium.

| **Genes** | **Biological implication of the graph** |
| --- | --- |
| **GILS3** | The GILS3 graph shows enrichment in Monarch for measurements related to serum alpha-1-antitrypsin, phosphatidylcholine, chloride, and creatine. |
| **ABCC4** | This graph highlights an enrichment in small molecules transportation within the apical membrane, specifically involving biological processes related to coumarin and urate metabolism. It also indicates molecular functions tied to the transmembrane transporter activity of urate and lipids, with 3 out of 5 genes involved in urate transport and 2 out of 8 genes involved in lipid transport. These findings collectively contribute to an enrichment in Monarch for both hypouricosuria and hypouricemia. |
| **SYNE1** | The graph shows enrichment in disease-associated gene databases for Donnai-Barrow syndrome and End-Stage Renal Disease (with 2 out of 2 and 2 out of 5 genes, respectively). Additionally, in Monarch, it is enriched for conditions such as low molecular weight proteinuria, abnormal glomerular mesangium morphology, and nephrotic syndrome. |
| **RREB1** | The graph demonstrates an enrichment in small molecules transportation across the apical membrane of the proximal tubule. It specifically involves biological processes related to export across the plasma membrane, xenobiotic transport, and urate metabolism. These findings collectively contribute to an enrichment in Monarch related to hyperuricemia. |
| **FANCC** | Process related to metabolism of macromolecules and DNA repair. |
| **TRPC1** | In KEGG this graph is enriched in inflammatory bowel disease and antifolate resistance. |
| **ABCA4** | Related to BBSome mediated cargo targeting cilium and cilium assembly in Reactome database. |
| **CDH20** | No significant enrichment |
| **E2F3** | This graph is related to signaling by interleukins and B cell receptors. In Monarch, it is enriched for abnormal biliary tract morphology, glomerular sclerosis, and exocrine pancreatic insufficiency. |
| **EPHA6** | This graph has been enriched for molecular function of transmembrane ephrin receptors. |
| **ENDOD1** | No significant enrichment. |
| **CEP128** | No significant enrichment. |
| **CCDC170** | No significant enrichment. |
| **CACNG3** | Voltage calcium channel complex. |
| **AJAP1** | In Monarch, it is associated with measurements of blood urea nitrogen, creatinine, glomerular filtration rate (GFR), creatine, and cystatin C. |
| **LAMA4** | In Monarch, it is involved in the measurement of blood urea nitrogen, creatinine measurement, GFR, creatine measurement, and cystatin C measurement. |
| **MAGI2** | The graph indicates enrichment in disease-associated gene databases for Donnai-Barrow syndrome and End-Stage Renal Disease. Additionally, these genes contained 2 of the 3 genes expressed in the renal proximal tubule epithelium. |
| **ABCA13** | No significant enrichment. |
